# Comparative Study on Flux Solution Methods of Discrete Unified Gas Kinetic Scheme

**DOI:** 10.3390/e27050528

**Published:** 2025-05-15

**Authors:** Wenqiang Guo

**Affiliations:** 1 National Key Laboratory of Aerospace Physics in Fluids, Mianyang 621000, China; guowenstrong@163.com; 2 Hypervelocity Aerodynamics Institute, China Aerodynamics Research and Development Center, Mianyang 621000, China

**Keywords:** discrete unified gas kinetic scheme, flux solution, Simpson method

## Abstract

In this work, the Simpson method is proposed to calculate the interface flux of a discrete unified gas kinetic scheme (DUGKS) according to the distribution function at the node and the midpoint of the interface, which is noted by Simpson–DUGKS. Moreover, the optimized DUGKS and Simpson–DUGKS considering the force term are derived. Then, the original DUGKS, optimized DUGKS, and Simpson–DUGKS are compared and analyzed in theory. Finally, the numerical tests are performed under different grid numbers (*N*). In the steady unidirectional flow (Couette flow and Poiseuille flow), the three methods are stable under different Courant–Friedrichs–Lewy (CFL) numbers, and the calculated *L*_2_ errors are the same. In the Taylor–Green vortex flow, the *L*_2_ error of the optimized DUGKS is the smallest with respect to the analytical solution of velocity, but the *L*_2_ error of the optimized DUGKS is the largest with respect to the analytical solution of density. In the lid-driven cavity flow, the results of the optimized DUGKS deviate more from the reference results in terms of accuracy, especially in the case of a small grid number. In terms of computational efficiency, it should be noted that the computational time of optimized DUGKS increases by about 40% compared with the original DUGKS when CFL = 0.1 and *N* = 16, and the calculation time of Simpson–DUGKS is reduced by about 59% compared with the original DUGKS when CFL = 0.95 and *N* = 16.

## 1. Introduction

Multiscale flows, characterized by significant spatial and temporal variations, are prevalent in natural phenomena and industrial processes. These flows pose substantial challenges to unified modeling and computational frameworks due to involving a large span of space and time [1]. In recent years, numerical methods for multiscale flow simulation have gained considerable attention across diverse fields, including micro–nano device design, aerospace engineering, and subsurface resource extraction [2,3,4,5,6,7,8,9]. For instance, during spacecraft atmospheric re-entry, the transition from continuum to rarefied flow regimes necessitates multiscale analysis [10,11,12]. While the Navier–Stokes equations, based on continuum assumptions, fail in rarefied regimes, the Boltzmann equation—derived from molecular dynamics principles—provides a more robust foundation. Numerical approaches to solving the Boltzmann equation include the following three categories: Particle-based methods decouple the transport and collision terms of the Boltzmann equation, simulating molecular streaming and collisions through computational particles. The direct simulation Monte Carlo (DSMC) method is a prominent example [13,14,15]. Discrete velocity methods (DVM) include the gas-kinetic unified algorithm (GKUA) [16], unified gas-kinetic scheme (UGKS) [17], and discrete unified gas kinetic scheme (DUGKS) [18]. By concurrently addressing transport and collision effects in flux calculations, these methods eliminate restrictions on the grid size and time steps imposed by molecular mean-free paths and collision times. This enables unified simulations across all flow regimes, overcoming DSMC’s limitations in continuum and near-continuum flows. In addition to stochastic particle methods and deterministic approaches, it would be valuable to include discussions on coupled methods. For example, under the UGKS framework, the unified gas-kinetic wave-particle (UGKWP) method demonstrates notable advantages in both computational efficiency and multiscale simulations across different flow regimes [19,20,21].

DUGKS distinguishes itself from the lattice Boltzmann method (LBM) and UGKS in several ways. While LBM primarily targets Navier–Stokes solutions for continuum flows and struggles in rarefied regimes, DUGKS achieves full flow-regime coverage across all Knudsen (Kn) numbers, capturing phenomena inaccessible to LBM [22,23]. As a finite-volume scheme, DUGKS naturally accommodates unstructured grids, enhancing geometric flexibility. Compared with UGKS, which relies on analytical time-integrated flux solutions, DUGKS employs a simplified numerical characteristic approach, reducing computational costs to approximately 70% of UGKS [24], while maintaining accuracy. DUGKS has been successfully applied to a wide range of multiscale flow problems, including turbulent flows [25,26,27,28], microscale flows (e.g., Knudsen layer effects, velocity slip) [29,30,31,32,33], compressible flows (shock waves, high-speed aerodynamics) [34,35,36,37], multiphase and particle-laden flows [38,39,40,41,42], complex geometries (3D unstructured meshes, porous media) [43,44,45,46,47,48,49], plasma dynamics and gas mixtures [45,46,50,51], and multiscale transport phenomena (phonon heat transfer [52,53,54], radiative transport [55,56]).

However, Wang et al. [57] think that the computational efficiency of DUGKS is not satisfactory for the following reasons: (1) The time step is determined by a small Courant–Friederichs–Lewy (CFL) number, which limits the overall computational efficiency. (2) The DUGKS framework is somewhat complicated due to the distribution function at the cell interface, which reduces the computational efficiency. Wang et al. [22] found that DUGKS is four times slower than LBM in the simulation of two-dimensional incompressible flow with the same grid resolution. Therefore, Wang et al. [57] proposed an optimized DUGKS to improve the efficiency of the original DUGKS. The evolution equation of the optimized DUGKS is approximately the same as that of the original DUGKS. Different from the original DUGKS, the optimized DUGKS uses a trapezoidal method to calculate the interface flux according to the distribution function at the nodes. However, there are higher-order methods to calculate interface flux than the trapezoidal method. Therefore, the Simpson method is proposed to calculate the interface flux according to the distribution function at the node and the midpoint of the interface, which is called Simpson–DUGKS in this paper. Moreover, the optimized DUGKS and Simpson–DUGKS considering force terms are derived. Then, the original DUGKS, optimized DUGKS, and Simpson–DUGKS are simply compared and analyzed in theory. Finally, the performance of original DUGKS, optimized DUGKS, and Simpson–DUGKS in computational efficiency, accuracy, and stability is tested by numerical examples, and a conclusion is drawn.

## 2. Numerical Method

### 2.1. Simplified Governing Equations of DUGKS with Force Term

Based on kinetic theory of gases, Guo et al. proposed the original DUGKS [18]. The basic variable is particle distribution function $f=f(\xi_{\alpha },x,t)$ for molecules moving with velocity $\xi_{\alpha }$ at position x and time t. Derived from the Boltzmann equation, the governing equation with force term DUGKS is as follows [23]:

(1) $$\frac{\partial f}{\partial t}+\xi_{\alpha }\cdot \nabla f=\Omega +S$$

Equation (1) describes the spatial and temporal evolution process of particle distribution function $f(\xi_{\alpha },x,t)$. The collision term Ω approximated by the Bhatnagar–Gross–Krook (BGK) model is as follows:

(2) $$\Omega =(f^{eq}-f)/\tau$$

The relaxation time τ is computed from τ=ν/RT, where ν, *R*, and *T* represent the kinematic viscosity, the gas constant, and the temperature, respectively.

The force term *S* is approximately [23]:

(3) $$S=-a\cdot \nabla_{\xi }f\approx a\cdot (\xi_{\alpha }-u)f^{eq}/RT$$

where a is the acceleration caused by the force term. In isothermal flow, the temperature is constant with $RT=c_{s}^{2}=1/3$.

At a low Mach number, Taylor expansion is carried out around zero particle velocity, and the Maxwell equilibrium distribution function *f^eq^* is approximately [18]:

(4) $$f^{eq}=W_{\alpha }\rho \left[1+\frac{\xi_{\alpha }\cdot u}{c_{s}^{2}}+\frac{{\left(\xi_{\alpha }\cdot u\right)}^{2}}{2c_{s}^{4}}-\frac{{\left|u\right|}^{2}}{2c_{s}^{2}}\right],\;c_{s}^{2}=RT.$$

The symbol $c_{s}$ represents the lattice speed of sound, which is a constant depending on the lattice arrangement ($c_{s}=1/\sqrt{3}$ for the D2Q9 model). The symbol *ρ* represents the macroscopic density.

In the D2Q9 (two-dimensional and nine velocity) model [55], the velocity space set {$\xi_{\alpha }$} and the corresponding weight set {$W_{\alpha }$} are as follows:

(5) $$\xi_{\alpha }=\left\{\begin{array}{l} \left(0,0\right)\;\;\;\;\;\;\;\;\;\;\;\;\;\;\alpha =0,\\ \left(\cos\left[\left(\alpha -1\right)\pi /2\right],\sin\left[\left(\alpha -1\right)\pi /2\right]\right)c,\;\;\;\alpha =1,2,3,4,\\ \left(\cos\left[\left(2\alpha -9\right)\pi /4\right],\sin\left[\left(2\alpha -9\right)\pi /4\right]\right)\sqrt{2}c,\;\alpha =5,6,7,8, \end{array}\right.\begin{array}{ccc} \begin{array}{c} \xi_{6}\\ ↖ \end{array} & \begin{array}{c} \xi_{2}\\ ↑ \end{array} & \begin{array}{c} \xi_{5}\\ ↗ \end{array}\\ \xi_{3}\leftarrow & \xi_{0} & \to \xi_{1}\\ \begin{array}{c} ↙\\ \xi_{7} \end{array} & \begin{array}{c} ↓\\ \xi_{4} \end{array} & \begin{array}{c} ↘\\ \xi_{8} \end{array} \end{array}$$

(6) $$W_{\alpha }=\left\{\begin{array}{l} \frac{4}{9},\;\;\;\alpha =0\\ \frac{1}{9},\;\;\;\alpha =1,2,3,4,\\ \frac{1}{36},\;\;\;\alpha =5,6,7,8, \end{array}\right.$$

The symbol *c* is the basic speed on the lattice (*c* = 1 usually).

**Figure 1:**
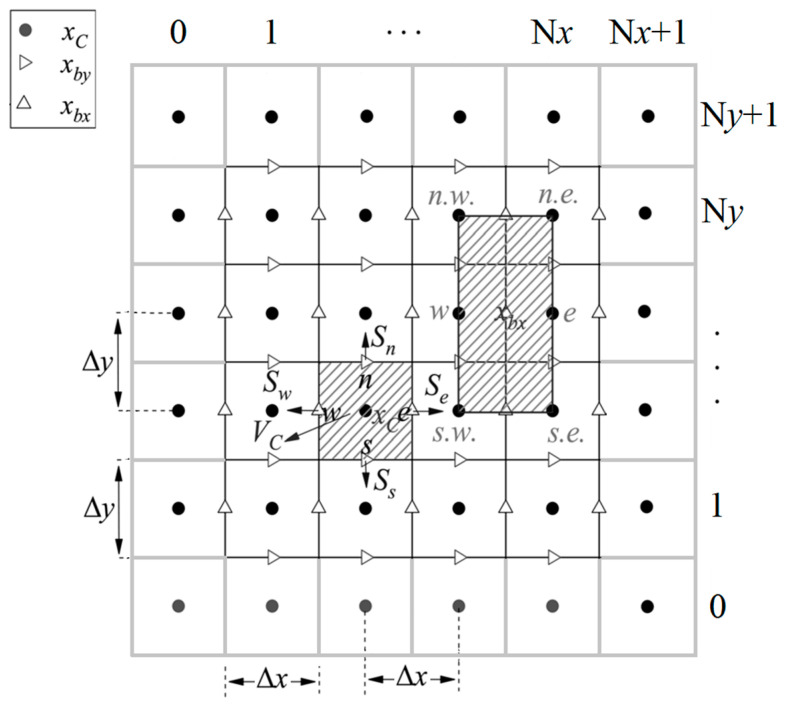
Schematic of two-dimensional uniform grid (Δx=Δy) of original DUGKS. It is noted that cells with *i* = 0 or N*x*+1, *j* = 0 or N*y*+1 are ghost cells, where the distribution function ${\bar{f}}_{C}^{+}$ at the center is obtained by the extrapolation and interpolation of adjacent cells.

In DUGKS with the D2Q9 model, the computational domain is divided into many cells by a Cartesian grid, and each cell has the size *V_C_* = Δ*x*Δ*y*, whose center is *x_C_*, as shown in Figure 1. As the finite volume scheme, the cell-average value of the distribution function and the force term are introduced.

(7) $$f_{C}^{n}=\frac{1}{\left|V_{C}\right|}\int_{V_{C}}f\left(\xi_{\alpha },x,t^{n}\right)dV,\;\;S_{C}^{n}=\frac{1}{\left|V_{C}\right|}\int_{V_{C}}S\left(\xi_{\alpha },x,t^{n}\right)dV$$

The subscript *C* represents the location in the cell center, and the superscript *n* represents the time *t^n^*. At each cell *V_C_*, Equation (1) is integrated from time *t^n^ = n*Δ*t* to time *t^n^*^+1^ *=* (*n* + 1)Δ*t*. The midpoint method is used to calculate the time integrals of the convection term, and the trapezoidal method is used to calculate the time integrals of the collision term and force term. The simplified governing equation of DUGKS is as follows

(8) $$\underset{{\tilde{f}}_{C}^{n+1}}{\underset{︸}{f_{C}^{n+1}-\frac{\Delta t}{2}\Omega_{C}^{n+1}-\frac{\Delta t}{2}S_{C}^{n+1}}}=\underset{{\tilde{f}}_{C}^{+,n}}{\underset{︸}{f_{C}^{n}+\frac{\Delta t}{2}\Omega_{C}^{n}+\frac{\Delta t}{2}S_{C}^{n}}}-\Delta tFlux^{n+1/2}$$

The following new transformation functions are introduced:

(9) $${\tilde{f}}_{C}\equiv f_{C}-\frac{\Delta t}{2}\Omega_{C}-\frac{\Delta t}{2}S_{C},\;\;{\tilde{f}}_{C}^{+}\equiv f_{C}+\frac{\Delta t}{2}\Omega_{C}+\frac{\Delta t}{2}S_{C}$$

Expanding the collision term in the BGK model, Equation (9) becomes as follows:

(10) $$f_{C}=\frac{2\tau }{2\tau +\Delta t}{\tilde{f}}_{C}+\frac{\Delta t}{2\tau +\Delta t}{f_{C}}^{eq}+\frac{\tau \Delta t}{2\tau +\Delta t}S_{C}$$

(11) $${\tilde{f}}_{C}^{+}=\frac{2\tau -\Delta t}{2\tau +\Delta t}{\tilde{f}}_{C}+\frac{2\Delta t}{2\tau +\Delta t}{f_{C}}^{eq}+\frac{2\tau \Delta t}{2\tau +\Delta t}S_{C}$$

The flux $Flux^{n+1/2}$ across the cell interface is calculated as follows:

(12) $$Flux^{n+1/2}=\frac{1}{\left|V_{C}\right|}\int_{\partial V_{C}}\left(\xi_{\alpha }\cdot n\right)f(\xi_{\alpha },x,t^{n+1/2})dS$$

It should be pointed out that in this paper, the calculation of interface flux is based on the distribution function at the time $t^{n+1/2}=t^{n}+h\;(h=\Delta t/2)$.

### 2.2. Three Methods for Calculating Interface Flux

#### 2.2.1. Original DUGKS

For the original DUGKS [18], the flux is calculated according to the midpoint method, as follows:

(13) $$Flux^{n+1/2}=\frac{1}{\left|V_{C}\right|}\int_{\partial V_{C}}\left(\xi_{\alpha }\cdot n\right)f(\xi_{\alpha },x_{b},t^{n+1/2})dS$$

***n*** is the outward unit vector normal to the cell interface ∂*V_C_*. $f(\xi_{\alpha },x_{b},t^{n+1/2})$ represents the distribution function of midpoint ***x****_b_* in the cell interface at the time $t^{n+1/2}=t^{n}+h\;(h=\Delta t/2)$.

Within half a time step (*t^n^* to *t^n^*^+1/2^), Equation (1) is integrated along the characteristic line (the midpoint ***x****_b_* of the cell interface is regarded as the end point of the characteristic line), and the collision term and force term are treated by the trapezoidal method, thus obtaining the following:

(14) $$\begin{array}{l} f\left(\xi_{\alpha },x_{b},t^{n+1/2}\right)-f\left(\xi_{\alpha },x_{b}-h\xi_{\alpha },t^{n}\right)=\\ \frac{h}{2}\left[\Omega \left(\xi_{\alpha },x_{b},t^{n+1/2}\right)+\Omega \left(\xi_{\alpha },x_{b}-h\xi_{\alpha },t^{n}\right)\right]+\frac{h}{2}\left[S\left(\xi_{\alpha },x_{b},t^{n+1/2}\right)+S\left(\xi_{\alpha },x_{b}-h\xi_{\alpha },t^{n}\right)\right] \end{array}$$

Similarly, the following new transformation functions are introduced:

(15) $$\bar{f}=f-\frac{h}{2}\Omega -\frac{h}{2}S=\frac{2\tau +h}{2\tau }f-\frac{h}{2\tau }f^{eq}-\frac{h}{2}S$$

(16) $${\bar{f}}^{+}=f+\frac{h}{2}\Omega +\frac{h}{2}S=\frac{2\tau -h}{2\tau }f+\frac{h}{2\tau }f^{eq}+\frac{h}{2}S$$

Then, Equation (14) can be simplified as follows:

(17) $$\bar{f}\left(\xi_{\alpha },x_{b},t^{n+1/2}\right)={\bar{f}}^{+}\left(\xi_{\alpha },x_{b}-h\xi_{\alpha },t^{n}\right)$$

Using Taylor expansion, the right term of Equation (17) can be approximated as follows:

(18) $${\bar{f}}^{+}\left(\xi_{\alpha },x_{b}-h\xi_{\alpha },t^{n}\right)={\bar{f}}^{+}\left(\xi_{\alpha },x_{b},t^{n}\right)-h\xi_{\alpha }\cdot \nabla {\bar{f}}^{+}\left(\xi_{\alpha },x_{b},t^{n}\right)$$

The auxiliary function ${\bar{f}}_{\alpha }^{+,n}$ at the midpoint of the interface and its gradient $\nabla {\bar{f}}_{\alpha }^{+,n}$ can be calculated by linear interpolation approximation, for example for the following directional uniform grid:

(19) $$\begin{array}{l} {\bar{f}}^{+}\left(\xi_{\alpha },x_{b},t^{n}\right)\approx {\bar{f}}^{+}\left(\xi_{\alpha },x_{C(i)},t^{n}\right)+\nabla {\bar{f}}^{+}\left(\xi_{\alpha },x_{b},t^{n}\right)\frac{\Delta x}{2}\\ \nabla {\bar{f}}^{+}\left(\xi_{\alpha },x_{b},t^{n}\right)\approx \frac{{\bar{f}}^{+}\left(\xi_{\alpha },x_{C(i+1)},t^{n}\right)-{\bar{f}}^{+}\left(\xi_{\alpha },x_{C(i)},t^{n}\right)}{\Delta x} \end{array}$$

The function ${\bar{f}}_{C}^{+}$ at the cell center ***x****_C_* can be obtained by the following transformation:

(20) $${\bar{f}}_{C}^{+}=\frac{2\tau -h}{2\tau +\Delta t}{\tilde{f}}_{C}+\frac{3h}{2\tau +\Delta t}{f_{C}}^{eq}+\frac{3\tau h}{2\tau +\Delta t}S_{C}$$

$\bar{f}(\xi_{\alpha },x_{b},t^{n+1/2})$ can be calculated according to Equations (17)–(20). Based on the conservation of mass and momentum, the density and velocity at the center of the cell interface are as follows:

(21) $${\left.\rho^{n+1/2}\right|}_{x_{b}}=\sum_{\alpha }\bar{f}\left(\xi_{\alpha },x_{b},t^{n+1/2}\right),{\left.{\left(\rho u\right)}^{n+1/2}\right|}_{x_{b}}=\sum_{\alpha }\xi_{\alpha }\bar{f}\left(\xi_{\alpha },x_{b},t^{n+1/2}\right)+0.5\rho ah$$

$f^{eq}$ is obtained according to Equation (4). $f(\xi_{\alpha },x_{b},t^{n+1/2})$ is determined according to Equation (15), and then, the flux $Flux^{n+1/2}$ can be obtained according to Equation (13). Then, according to Equation (8), the distribution function ${\tilde{f}}_{C}$ tracked in the DUGKS can be updated to the next time step. Since ${\bar{f}}_{C}^{+}$ has been obtained according to Equation (20), ${\tilde{f}}_{C}^{+}$ is obtained by the following equation:

(22) $${\tilde{f}}_{C}^{+}=\frac{4}{3}{\bar{f}}_{C}^{+}-\frac{1}{3}{\tilde{f}}_{C}$$

The evolution steps of the original DUGKS to update to the next time step are as follows:

$$\begin{array}{l} {\tilde{f}}_{C}^{n}\overset{\left(20),(22\right)}{\to }{\bar{f}}_{C}^{+,n},{\tilde{f}}_{C}^{+,n}\overset{\left(17-19\right)}{\to }\bar{f}\left(\xi_{\alpha },x_{b},t^{n+1/2}\right)\\ \overset{\left(21),(4\right)}{\to }f^{eq}\left(\xi_{\alpha },x_{b},t^{n+1/2}\right)\overset{\left(15\right)}{\to }f\left(\xi_{\alpha },x_{b},t^{n+1/2}\right)\\ \overset{\left(13\right)}{\to }Flux^{n+1/2}\overset{\left(8\right)}{\to }{\tilde{f}}_{C}^{n+1} \end{array}$$

In this paper, for the original DUGKS, optimized DUGKS, and Simpson–DUGKS, the macroscopic quantity (density and velocity) at the cell center can be obtained by tracking the following distribution function ${\tilde{f}}_{C}$:

(23) $$\rho =\sum_{\alpha }{\tilde{f}}_{C}^{n},\;\;\rho u=\sum_{\alpha }\xi_{\alpha }{\tilde{f}}_{C}^{n}+0.5\rho a\Delta t$$

In all flow regimes, the time step Δt is independent of the relaxation time τ and is determined by the Courant–Friedrichs–Lewy (CFL) condition [18]:

(24) $$\Delta t=CFL\frac{\Delta x_{\min}}{C}$$

$\Delta x_{\min}$ represents the minimum grid spacing. The maximum discrete speed C is set to C = $\sqrt{2}$. The CFL number is defined as *CFL* = *C*Δ*t*/Δ*x*_min_. The CFL condition ensures that information propagating through the computational domain (e.g., waves or disturbances) does not travel further than one grid cell per time step. If the CFL condition is violated (i.e., the CFL number is too large), numerical instabilities can arise, leading to non-physical oscillations or the divergence of the solution. As demonstrated by the authors of [58], the stability of DUGKS is not affected by the CFL number apparently as long as CFL < 1. So, the CFL number often lies in 0 and 1.

Taking the two-dimensional uniform grid as an example, as shown in Figure 1, the evolution process of the distribution function of the original DUGKS can be written as follows:

(25) $$\begin{array}{l} {\tilde{f}}_{C}^{n+1}={\tilde{f}}_{C}^{+,n}-\Delta t\left[\frac{\xi_{x}}{\Delta x}\left(f_{be}^{n+1/2}-f_{bw}^{n+1/2}\right)+\frac{\xi_{y}}{\Delta y}\left(f_{bn}^{n+1/2}-f_{bs}^{n+1/2}\right)\right]\\ =\frac{4{\bar{f}}_{C}^{+,n}-{\tilde{f}}_{C}^{n}}{3}-\Delta t\sum_{\left(i\right)}\psi_{\left(i\right)}f_{b(i)}^{n+1/2} \end{array}.$$

where $\psi_{e}=-\psi_{w}=\xi_{x}/\Delta x$, $\psi_{n}=-\psi_{s}=\xi_{y}/\Delta y$. The distribution function $f(\xi_{\alpha },x_{b},t^{n+1/2})$ is abbreviated as $f_{b}^{n+1/2}$. The distribution function ${\bar{f}}_{bx}^{n+1/2}$ at the midpoint *x_bx_* of the cell interface can be obtained by interpolating the distribution functions of the surrounding six cell centers:

(26) $$\begin{array}{l} {\bar{f}}_{bx}^{n+1/2}=\left(\frac{1}{2}-\frac{\Delta t}{2}\frac{\xi_{x}}{\Delta x}\right){\bar{f}}_{Ce}^{+,n}+\left(\frac{1}{2}+\frac{\Delta t}{2}\frac{\xi_{x}}{\Delta x}\right){\bar{f}}_{Cw}^{+,n}-\frac{\Delta t\xi_{y}}{8\Delta y}\left({\bar{f}}_{Cn.e.}^{+,n}+{\bar{f}}_{Cn.w.}^{+,n}-{\bar{f}}_{Cs.e.}^{+,n}-{\bar{f}}_{Cs.w.}^{+,n}\right)\\ =\sum_{\left(i\right)}\left(\omega_{\left(i\right)}-\frac{\Delta t}{2}\psi_{\left(i\right)}\right){\bar{f}}_{C(i)}^{+,n} \end{array}.$$

where

$$\omega_{e}=\omega_{w}=1/2,\omega_{n.e.}=\omega_{n.w.}=\omega_{s.e.}=\omega_{s.w.}=0,\psi_{n.e.}=\psi_{n.w.}=\psi_{s.e.}=\psi_{s.w.}=\xi_{y}/4\Delta y.$$

Then, the evolution steps of ${\tilde{f}}_{C}$ updating to the next time step in the original DUGKS in the two-dimensional uniform grid are as follows:

(27) $$\begin{array}{l} {\tilde{f}}_{C}^{n}\overset{\left(20),(22\right)}{\to }{\bar{f}}_{C}^{+,n},{\tilde{f}}_{C}^{+,n}\overset{\left(26\right)}{\to }{\bar{f}}_{bx}^{n+1/2},{\bar{f}}_{by}^{n+1/2}\\ \overset{\left(21),(4\right)}{\to }f_{bx}^{eq,n+1/2},f_{by}^{eq,n+1/2}\overset{\left(15\right)}{\to }f_{bx}^{n+1/2},f_{by}^{n+1/2}\overset{\left(25\right)}{\to }{\tilde{f}}_{C}^{n+1} \end{array}$$

#### 2.2.2. Optimize DUGKS

For the optimized DUGKS [57], the trapezoidal method is used to calculate the interface flux:

(28) $$Flux^{n+1/2}=\frac{1}{\left|V_{C}\right|}\int_{\partial V_{C}}\left(\xi_{\alpha }\cdot n\right)\frac{f(\xi_{\alpha },x_{N(i)},t^{n+1/2})+f(\xi_{\alpha },x_{N(i+1)},t^{n+1/2})}{2}dS$$

$f(\xi_{\alpha },x_{N},t^{n+1/2})$ represents the distribution function at the intersection point (node) ***x****_N_* of the cell interface at the time $t^{n+1/2}=t^{n}+h\;(h=\Delta t/2)$, which is abbreviated as $f_{N}^{n+1/2}$.

**Figure 2:**
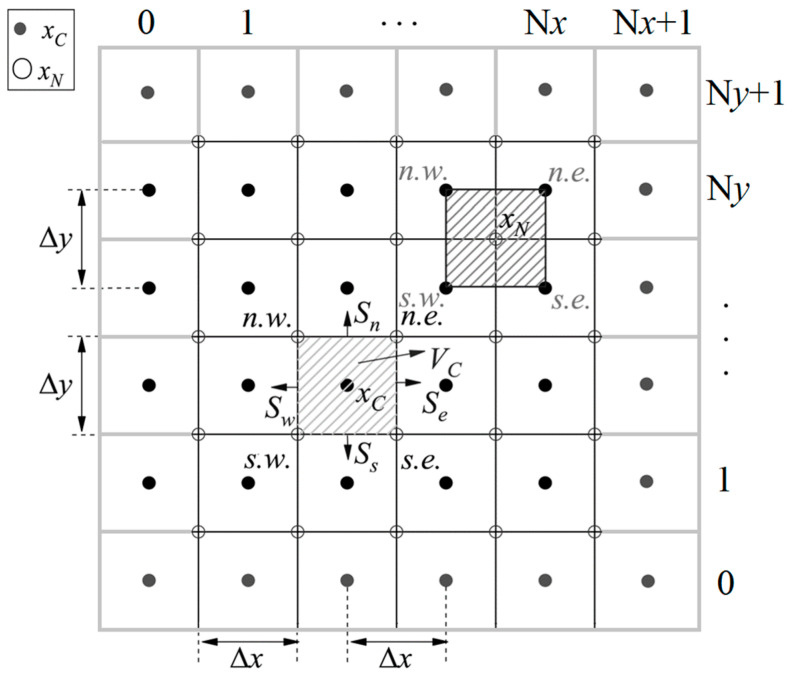
Schematic of two-dimensional uniform grid (Δx=Δy) of optimized DUGKS. It is noted that cells with *i* = 0 or N*x*+1, *j* = 0 or N*y*+1 are ghost cells, where the distribution function ${\bar{f}}_{C}^{+}$ at the center is obtained by the extrapolation and interpolation of adjacent cells.

Taking the two-dimensional uniform grid as an example, as shown in Figure 2, the calculation formula of interface flux $Flux^{n+1/2}$ in the optimized DUGKS is as follows:

(29) $$\begin{array}{l} Flux^{n+1/2}=\frac{\xi_{x}}{\left|V_{C}\right|}\left(\frac{f_{Nn.e.}^{n+1/2}+f_{Ns.e.}^{n+1/2}}{2}S_{e}-\frac{f_{Nn.w.}^{n+1/2}+f_{Ns.w.}^{n+1/2}}{2}S_{w}\right)\\ +\frac{\xi_{y}}{\left|V_{C}\right|}\left(\frac{f_{Nn.w.}^{n+1/2}+f_{Nn.e.}^{n+1/2}}{2}S_{n}-\frac{f_{Ns.w.}^{n+1/2}+f_{Ns.e.}^{n+1/2}}{2}S_{s}\right)=\sum_{\left(i\right)}\psi_{\left(i\right)}f_{N(i)}^{n+1/2}. \end{array}$$

where $\psi_{n.e.}=-\psi_{s.w.}=\left(\xi_{x}/\Delta x+\xi_{y}/\Delta y\right)/2$, $\psi_{s.e.}=-\psi_{n.w.}=\left(\xi_{x}/\Delta x-\xi_{y}/\Delta y\right)/2$.

Next, the calculation of distribution function $f_{N}^{n+1/2}$ is introduced.

Using Taylor expansion, $f_{N}^{n+1/2}$ can be obtained from Equation (1) (see the Appendix A) as follows:

(30) $$\underset{{\bar{f}}_{N}^{n+1/2}}{\underset{︸}{f_{N}^{n+1/2}-\frac{h}{2}\Omega_{N}^{n+1/2}-\frac{h}{2}S_{N}^{n+1/2}}}=\underset{{\bar{f}}_{N}^{+,n}}{\underset{︸}{f_{N}^{n}+\frac{h}{2}\Omega_{N}^{n}+\frac{h}{2}S_{N}^{n}}}-h\xi_{\alpha }\cdot \nabla {\bar{f}}_{N}^{+,n}.$$

${\bar{f}}_{N}^{+,n}$ and $\nabla {\bar{f}}_{N}^{+,n}$ can be obtained by the following distribution function of the surrounding four cell centers:

(31) $${\bar{f}}_{N}^{n+1/2}=\sum_{\left(i\right)}\omega_{\left(i\right)}{\bar{f}}_{C(i)}^{+,n}-\frac{\Delta t}{2}\sum_{\left(i\right)}\psi_{\left(i\right)}{\bar{f}}_{C(i)}^{+,n}=\sum_{\left(i\right)}\left(\omega_{\left(i\right)}-\frac{\Delta t}{2}\psi_{\left(i\right)}\right){\bar{f}}_{C(i)}^{+,n}.$$

where $\omega_{n.e.}=\omega_{n.w.}=\omega_{s.e.}=\omega_{s.w.}=1/4$.

$f_{N}^{n+1/2}$ can be calculated after ${\bar{f}}_{N}^{n+1/2}$ is known, as follows:

(32) $$f_{N}^{n+1/2}=\frac{2\tau }{2\tau +h}{\bar{f}}_{N}^{n+1/2}+\frac{h}{2\tau +h}f_{N}^{eq,n+1/2}+\frac{\tau h}{2\tau +h}S_{N}^{n+1/2}.$$

$f_{N}^{eq,n+1/2}$ is obtained from Equation (4), where the density and velocity are as follows:

(33) $${\left.\rho^{n+1/2}\right|}_{x_{N}}=\sum_{\alpha }{\bar{f}}_{N}^{n+1/2},\;\;{\left.{\left(\rho u\right)}^{n+1/2}\right|}_{x_{N}}=\sum_{\alpha }\xi_{\alpha }{\bar{f}}_{N}^{n+1/2}+0.5\rho ah$$

The evolution process of the distribution function of optimization DUGKS in the two-dimensional uniform grid can be written as follows:

(34) $${\tilde{f}}_{C}^{n+1}=\frac{4{\bar{f}}_{C}^{+,n}-{\tilde{f}}_{C}^{n}}{3}-\Delta t\sum_{\left(i\right)}\psi_{\left(i\right)}f_{N(i)}^{n+1/2}.$$

Then, the evolution steps of updating to the next time step in the optimization DUGKS are as follows:

(35) $${\tilde{f}}_{C}^{n}\overset{\left(20),(22\right)}{\to }{\bar{f}}_{C}^{+,n},{\tilde{f}}_{C}^{+,n}\overset{\left(31\right)}{\to }{\bar{f}}_{N}^{n+1/2}\overset{\left(33),(4\right)}{\to }f_{N}^{eq,n+1/2}\overset{\left(32\right)}{\to }f_{N}^{n+1/2}\overset{\left(34\right)}{\to }{\tilde{f}}_{C}^{n+1}.$$

#### 2.2.3. Simpson–DUGKS

For Simpson–DUGKS, the Simpson method (three-point method) is proposed to calculate the interface flux, as follows:

(36) $$Flux^{n+1/2}=\frac{1}{\left|V_{C}\right|}\int_{\partial V_{C}}\left(\xi_{\alpha }\cdot n\right)\frac{f(\xi_{\alpha },x_{N(i)},t^{n+1/2})+4f(\xi_{\alpha },x_{b},t^{n+1/2})+f(\xi_{\alpha },x_{N(i+1)},t^{n+1/2})}{6}dS$$

Taking the two-dimensional uniform grid as an example, as shown in Figure 2, the calculation formula of interface flux $Flux^{n+1/2}$ in the Simpson–DUGKS is as follows:

(37) $$\begin{array}{l} Flux^{n+1/2}=\frac{\xi_{x}}{\left|V_{C}\right|}\left(\frac{f_{Nn.e.}^{n+1/2}+4f_{be}^{n+1/2}+f_{Ns.e.}^{n+1/2}}{6}S_{e}-\frac{f_{Nn.w.}^{n+1/2}+4f_{bw}^{n+1/2}+f_{Ns.w.}^{n+1/2}}{6}S_{w}\right)\\ +\frac{\xi_{y}}{\left|V_{C}\right|}\left(\frac{f_{Nn.w.}^{n+1/2}+4f_{bn}^{n+1/2}+f_{Nn.e.}^{n+1/2}}{6}S_{n}-\frac{f_{Ns.w.}^{n+1/2}+4f_{bs}^{n+1/2}+f_{Ns.e.}^{n+1/2}}{6}S_{s}\right)\\ =\sum_{\left(i\right)}{\overset{Opt.}{\psi }}_{\left(i\right)}f_{N(i)}^{n+1/2}+\sum_{\left(j\right)}{\overset{Ori.}{\psi }}_{\left(j\right)}f_{b(i)}^{n+1/2} \end{array}$$

where ${\overset{Opt.}{\psi }}_{n.e.}=-{\overset{Opt.}{\psi }}_{s.w.}=\left(\xi_{x}/\Delta x+\xi_{y}/\Delta y\right)/6$, ${\overset{Opt.}{\psi }}_{s.e.}=-{\overset{Opt.}{\psi }}_{n.w.}=\left(\xi_{x}/\Delta x-\xi_{y}/\Delta y\right)/6$,${\overset{Ori.}{\psi }}_{e}=-{\overset{Ori.}{\psi }}_{w}=2\xi_{x}/3\Delta x$, ${\overset{Ori.}{\psi }}_{n}=-{\overset{Ori.}{\psi }}_{s}=2\xi_{y}/3\Delta y$.

The evolution process of the distribution function of Simpson–DUGKS in the two-dimensional uniform grid can be written as follows:

(38) $${\tilde{f}}_{C}^{n+1}=\frac{4{\bar{f}}_{C}^{+,n}-{\tilde{f}}_{C}^{n}}{3}-\Delta t\left(\sum_{\left(i\right)}{\overset{Opt.}{\psi }}_{\left(i\right)}f_{N(i)}^{n+1/2}+\sum_{\left(j\right)}{\overset{Ori.}{\psi }}_{\left(j\right)}f_{b(i)}^{n+1/2}\right).$$

Then, the evolution steps of updating to the next time step in the Simpson–DUGKS are as follows:

(39) $${\tilde{f}}_{C}^{n}\overset{\left(20),(22\right)}{\to }{\bar{f}}_{C}^{+,n},{\tilde{f}}_{C}^{+,n}\to \left[\begin{array}{c} \overset{\left(26\right)}{\to }{\bar{f}}_{bx}^{n+1/2},{\bar{f}}_{by}^{n+1/2}\overset{\left(21),(4\right)}{\to }f_{bx}^{eq,n+1/2},f_{by}^{eq,n+1/2}\overset{\left(15\right)}{\to }f_{bx}^{n+1/2},f_{by}^{n+1/2}\\ \overset{\left(31\right)}{\to }{\bar{f}}_{N}^{n+1/2}\overset{\left(33),(4\right)}{\to }f_{N}^{eq,n+1/2}\overset{\left(32\right)}{\to }f_{N}^{n+1/2} \end{array}\right)\overset{\left(38\right)}{\to }{\tilde{f}}_{C}^{n+1}.$$

### 2.3. Comparative Analysis

As shown in Figure 1 and Figure 2, the number of the nodes *x_N_* is (N*x*+1) (N*y*+1), the number of *x_bx_* in the *x* direction is N*y* (N*x*+1), the number of *x_by_* in the *y* direction is N*x* (N*y*+1), and the number of *x_C_* is N*x*N*y*. In the original DUGKS, the flux is calculated according to the distribution function at the cell interface center ***x****_b_*. In the optimized DUGKS, the flux is calculated according to the distribution function at the intersection point (node) ***x****_N_*. In the Simpson–DUGKS, the flux is calculated according to the distribution function of ***x****_b_* and ***x****_N_*. Therefore, the total number of points is N*x*N*y* + N*y* (N*x*+1) + N*x* (N*y*+1) in the original DUGKS, the total number of points is N*x*N*y* + (N*x*+1) (N*y*+1) in the optimized DUGKS, and the total number of points is 4N*x*N*y*+2 (N*x* + N*y*) + 1 in the Simpson–DUGKS. It can be seen that the optimized DUGKS has the minimum computational cost, so the optimized DUGKS has the highest computational efficiency in theoretical analysis.

There are three methods to evaluate the interface flux, as follows: the original DUGKS adopts the midpoint method; the optimize DUGKS adopts the trapezoidal method; and Simpson–DUGKS adopts the Simpson method (three-point method). The truncation errors of these three integration methods are as follows:

(40) $$\int_{x_{A}}^{x_{B}}f(x)dx=\left\{\begin{array}{c} \left(x_{B}-x_{A})f\left(\frac{x_{A}+x_{B}}{2}\right)+\frac{1}{24}{\left(x_{B}-x_{A}\right)}^{3}f^{\prime \prime }(\xi ),\;\xi \in [x_{A},x_{B}\right]\\ \left(x_{B}-x_{A})\frac{f(x_{A})+f(x_{B})}{2}-\frac{1}{12}{\left(x_{B}-x_{A}\right)}^{3}f^{\prime \prime }(\xi \right)\\ \left(x_{B}-x_{A})\frac{f(x_{A})+4f\left(\frac{x_{A}+x_{B}}{2}\right)+f(x_{B})}{6}-\frac{1}{2880}{\left(x_{B}-x_{A}\right)}^{5}f^{\left(4\right)}(\xi \right) \end{array}\right..$$

From Equation (40), it can be seen that the local truncation error of midpoint method is twice that of trapezoidal method. The algebraic accuracy of midpoint method and trapezoidal method is 1, and the algebraic accuracy of Simpson method is 3. Both the midpoint method and the trapezoidal method introduce spurious dissipation due to integration errors, smoothing out small-scale vortex structures. Moreover, they may also distort the phase characteristics of waves, affecting the simulation of transient flows. The Simpson method reduces these errors by means of high-order polynomial interpolation and lower truncation errors, and it has a significant effect, especially when simulating problems, such as unsteady and transient flows.

In the next section, the computational efficiency and error of these three methods are compared by numerical tests, as well as the stability.

## 3. Numerical Test

The Couette flow, Poiseuille flow, Taylor–Green vortex flow, and lid-driven cavity flow are numerically tested. In the simulations, Δx=Δy=1/Nx=1/Ny=1/N, $\Delta t=\mathrm{CFL}*\Delta x/\sqrt{2}$, τ=υ/RT=3υ, and *ν* = *HU*_0_/Re. The characteristic height *H* is fixed as *H* = 1, and the reference velocity *U*_0_ is fixed as *U*_0_ = 0.1. The numerical experiments demonstrate that the maximum CFL number available for original and optimized DUGKS are around 1.0 and 1.7 [57], and the CFL number often lies in 0 and 1.0. Therefore, the CFL number is selected as CFL = 0.1, 0.5, 0.95, 1.0, 1.3, 1.5, and 1.7 in this work. Considering the computational cost, the grid size including the low-resolution grid, medium-resolution grid, and high-resolution grid is selected as *N* = 16, 32, 64, and 128. The grid number *N*, CFL number, and Re number are adjusted to compare the computational efficiency, accuracy, and numerical stability under different conditions. To compare the numerical stability, the ratio of time step to relaxation time (∆*t*/*τ* = 5√2*CFL*Re/(3N)) is considered. So, a high Re number (Re = 10,000) is selected for the numerical tests, and some commonly used Re number (Re = 100 and 1000) is also selected.

The convergence criteria for attaining the steady-state solution are defined as follows:

(41) $$error=\sum_{i,j}\left|u_{ij}^{n}-u_{ij}^{n-1000}\right|/\sum_{i,j}\left|u_{ij}^{n}\right|\leq 10^{-6}$$

where $u_{ij}^{n}=u(x_{i},y_{j},n\Delta t)$ represents the velocity in the fluid domain at time *t* = *n*Δ*t*. *error* represents the convergence error. Unless stated otherwise, the convergence criterion is set to be *error* < 10^−6^.

To assess the accuracy, the *L*_2_ error of macroscopic physical quantity is estimated as follows:

(42) $$L_{2}err=\sqrt{\sum (u-u^{\prime }{)}^{2}}/\sqrt{\sum (u^{\prime }{)}^{2}}$$

$u^{\prime }$ is the analytical solution.

To keep the boundary conditions consistent when comparing the three methods, the non-slip solid wall boundary condition adopts a bounce-back scheme to deal with the distribution function ${\bar{f}}_{C}^{+}$ at the center of the ghost cells (*i* = 0 or N*x*+1, *j* = 0 or N*y*+1), as shown in Figure 1 and Figure 2.

It should be noted that all the simulations are performed on an Intel(R) Core(TM) i9-10900K CPU and 64-bit operating system.

### 3.1. Couette Flow

In the Couette flow, the lower wall is fixed, and the upper wall moves with horizontal velocity *U*_0_ = 0.1. Periodic boundary conditions are adopted for inlet and outlet (left and right boundaries). The analytical solution of horizontal velocity *u*(*y*) in the Couette flow is as follows:

(43) $$u(y)/U_{0}=y/H.$$

The CPU time, *L*_2_ error, and the number of steps to reach convergence calculated by the three methods are shown in Table 1. Note that the symbol “-” indicates that the calculation does not converge, Δ1 represents the percentage increase in the calculation time of Simpson–DUGKS compared with the original DUGKS, and Δ2 represents the percentage decrease in the calculation time of optimized DUGKS compared with the original DUGKS. In terms of accuracy, the *L*_2_ errors calculated by the three methods according to Equation (42) are the same. In terms of computational efficiency, although the three methods have the same number of steps to achieve the convergence criterion of Equation (41), the computational time of optimized DUGKS is reduced by about 18–47% compared with the original DUGKS, and the computational time of Simpson–DUGKS is increased by about 27–120% compared with the original DUGKS. In terms of computational stability, the three methods have the same stability under different CFL numbers and grid numbers in the test. All three methods can keep the computational convergence, except for the divergence at N*x* = N*y* = 128 with CFL = 1.7.

Furthermore, the numerical performance of the three methods with different CFL numbers (CFL = 0.95, 1.5, 1.7) and different grid numbers (N*x* = N*y* = 16, 32, 64, 128) is tested at a high Re number. The results are shown in Table 2. With CFL = 0.95 and 1.5, the three methods do not converge with the grid numbers N*x* = N*y* = 16, 32, and 64 but converge stably only with the grid number N*x* = N*y* = 128. With CFL = 1.7, the three methods cannot work with the grid number N*x* = N*y* = 16, 32, and 64. Only with the grid number N*x* = N*y* = 128, the calculation can converge stably. In terms of computational efficiency, although the steps to reach the convergence criterion of Equation (41) are the same among the three methods, the computational time of the optimized DUGKS is reduced by about 14–39% compared with that of the original DUGKS.

**Figure 3:**
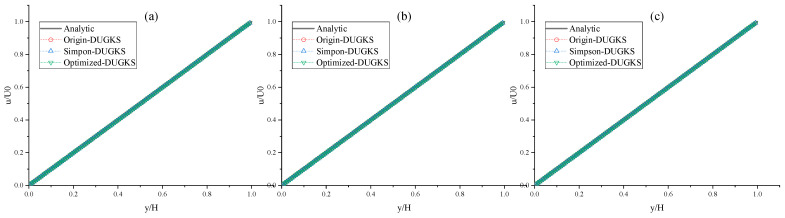
Horizontal velocity profiles at Re = 10,000 with the grid number N*x* = N*y* = 128. (**a**) CFL = 0.95, (**b**) CFL = 1.5, (**c**) CFL = 1.7.

In terms of accuracy, the *L*_2_ errors calculated by the three methods according to Equation (42) are the same. The corresponding velocity distribution is shown in Figure 3, which is in good agreement with the analytical solution.

### 3.2. Poiseuille Flow

The Poiseuille flow is driven by external force *ρa_x_* (*a_y_* = 0), and the inlet and outlet (left and right boundaries) adopt periodic boundary conditions. The analytical solution of the horizontal velocity *u*(*y*) in the Poiseuille flow can be expressed as follows:

(44) $$u(y)/U_{0}=4y/H\left(1-y/H\right).$$

where *U*_0_ = *a_x_H*^2^/8*ν*.

The CPU time, *L*_2_ error, and the number of steps to reach convergence calculated by the three methods are shown in Table 3. Note that Δ1 represents the percentage increase in the calculation time of Simpson–DUGKS compared with the original DUGKS, and Δ2 represents the percentage decrease in the calculation time of optimized DUGKS compared with the original DUGKS. In terms of accuracy, the *L*_2_ errors calculated by the three methods according to Equation (42) are the same. In terms of computational efficiency, although the three methods have the same number of steps to achieve the convergence criterion of Equation (41), the computational time of optimized DUGKS is reduced by about 20–39% compared with the original DUGKS, and the computational time of Simpson–DUGKS is increased by about 35–62% compared with the original DUGKS. In terms of computational stability, the three methods have the same stability under different CFL numbers and grid numbers in the test. The three methods can keep the computational convergence, except for the divergence with the grid number N*x* = N*y* = 128 and CFL = 1.7.

**Figure 4:**
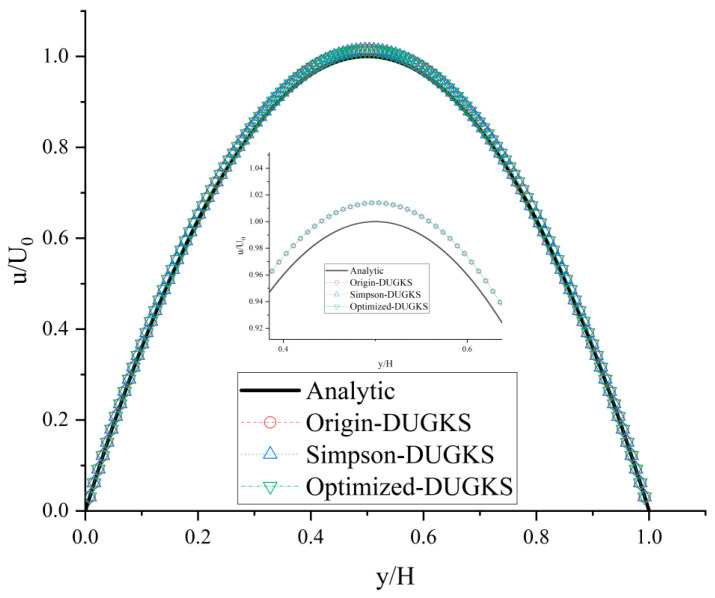
Horizontal velocity distribution at Re = 10,000 with CFL = 1.7 and N*x* = N*y* = 128.

Furthermore, the numerical performance of the three methods with different grid numbers is tested at high Re number and large CFL number, as shown in Table 4. With the grid number N*x* = N*y* = 16, 32, and 64, the three methods cannot work. Only with the grid number N*x* = N*y* = 128, the calculation can converge stably. In terms of computational efficiency, although the steps to reach the convergence criterion of Equation (41) are the same among the three methods, the computational time of the optimized DUGKS is reduced by about 44% compared with that of the original DUGKS. In terms of accuracy, the *L*_2_ errors calculated by the three methods according to Equation (42) are the same. The corresponding velocity distribution is shown in Figure 4, which is in agreement with the analytical solution. Zooming in on this area can show that the predictions of the three methods noticeably deviate from the theoretical values near the center.

### 3.3. Taylor–Green Vortex Flow

The Taylor–Green vortex flow is characterized by turbulence and unsteady in macroscopic view. It is a simple model for studying the decomposition process of larger-scale vortexes gradually declining into smaller-scale vortexes and the resulting uniform isotropic turbulence. It is widely used as a standard example of a numerical algorithm for fluid solutions. In the two-dimensional Taylor–Green vortex flow with periodic boundary conditions, the analytical solutions of its velocity and density are as follows:

(45) $$u(x,y,t)=-U_{0}\cos(k_{x}x)\sin(k_{y}y)e^{-k^{2}\upsilon t}.$$

(46) $$v(x,y,t)=\frac{k_{x}}{k_{y}}U_{0}\sin(k_{x}x)\cos(k_{y}y)e^{-k^{2}\upsilon t}.$$

(47) $$\rho (x,y,t)=\rho_{0}-\frac{1}{4}\rho_{0}\frac{U_{0}^{2}}{RT}\left[\cos(2k_{x}x)+{\left(\frac{k_{x}}{k_{y}}\right)}^{2}\cos(2k_{y}y)\right]e^{-2k^{2}\upsilon t}.$$

where *u* and *v* denote horizontal and vertical velocities, respectively. *ρ* denotes the density. $k_{x}=2\pi /L_{x}$ and $k_{y}=2\pi /L_{y}$ represent the wave numbers in the horizontal and vertical direction after expansion through Fourier space, respectively. $k=\sqrt{k_{x}^{2}+k_{y}^{2}}$ represents the wave number in Fourier space. *υ* denotes the kinematic viscosity of fluid. *U*_0_ denotes the initial velocity peak. *ρ*_0_ denotes the average density, and it is set as *ρ*_0_ = 1.

**Figure 5:**
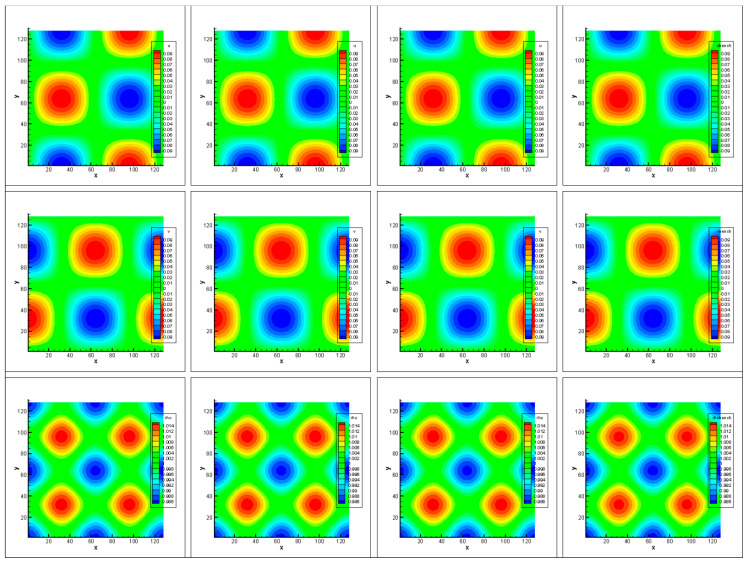
Velocity and density contours. Each row from top to bottom represents horizontal velocity, vertical velocity, and density, respectively. Each column from left to right represents the contours obtained by the original DUGKS, Simpson–DUGKS, and optimized DUGKS, respectively. The rightmost column represents the analytical solution.

**Figure 6:**
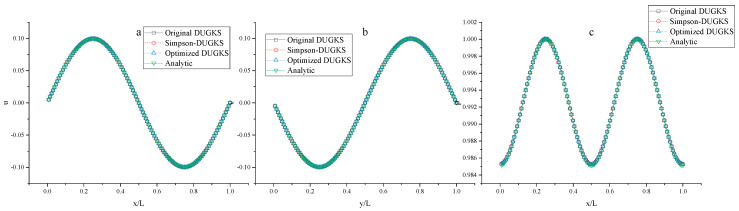
Velocity and density profiles. (**a**) Horizontal velocity *u*, (**b**) vertical velocity *v*, (**c**) and density *ρ.*

**Figure 7:**
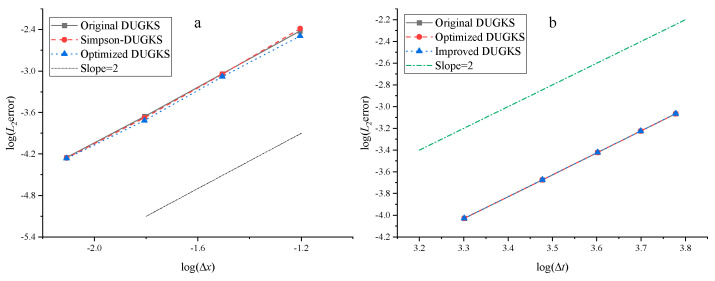
Errors of the velocity using different grid size Δ*x* (**a**) and varies Δ*t* (**b**).

The convergence error, *L*_2_ error, and CPU time (s) calculated by the three methods to 10,000 steps at Re = 1000 are tested, as shown in Table 5, Table 6, Table 7 and Table 8. Note that Δ1 represents the percentage increase in the calculation time of Simpson–DUGKS compared with the original DUGKS, and Δ2 represents the percentage decrease in the calculation time of optimized DUGKS compared with the original DUGKS. In terms of computational stability, all three methods can calculate stably under different grid numbers in the test. In terms of accuracy, all three methods are in good agreement with the analytical solution, as shown in Figure 5 and Figure 6. However, as shown in Table 5, Table 6 and Table 7, the convergence error calculated by Equation (41) and the *L*_2_ error calculated by Equation (42) are different. The convergence error of optimized DUGKS is the smallest under the same number of grids. Compared with the analytical solution of velocity, the *L*_2_ error of the optimized DUGKS is the smallest, and the *L*_2_ error of the original DUGKS is the largest under the same grid number. Compared with the analytical solution of density, the *L*_2_ error of optimized DUGKS is the largest under the same number of grids. As shown in Table 8, in terms of computational efficiency, when the number of calculation steps is 10,000, the calculation time of optimized DUGKS is reduced by about 12–24% compared with the original DUGKS, and the calculation time of Simpson–DUGKS is increased by about 22–30% compared with the original DUGKS. As shown in Figure 7, all three methods are of second-order accuracy in space and time.

### 3.4. Lid-Driven Cavity Flow

As a standard example, the lid-driven cavity flow contains complex flows, and the results of Ghia et al. [59] are usually used as reference data. The upper wall moves with horizontal velocity *U*_0_ = 0.1, and the lower wall and left- and right-side walls are fixed.

**Figure 8:**
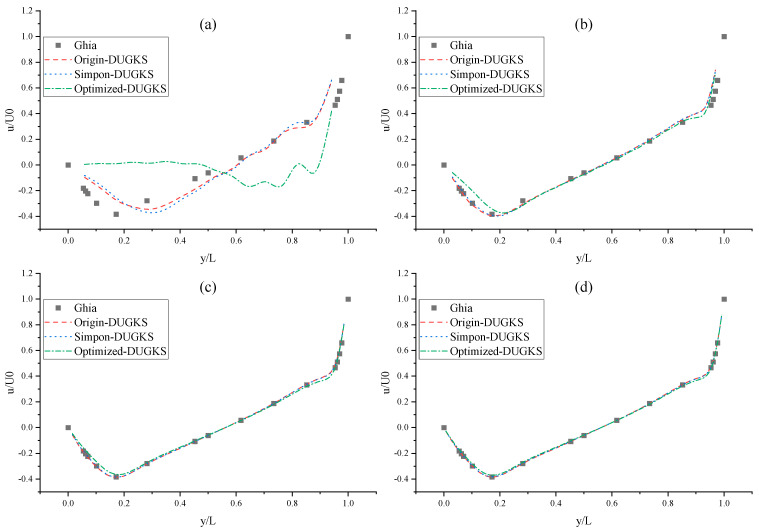
Horizontal velocity distribution of vertical central axis at Re = 1000 (CFL = 0.1). (**a**) N = 16, (**b**) N = 32, (**c**) N = 64, (**d**) N = 128.

**Figure 9:**
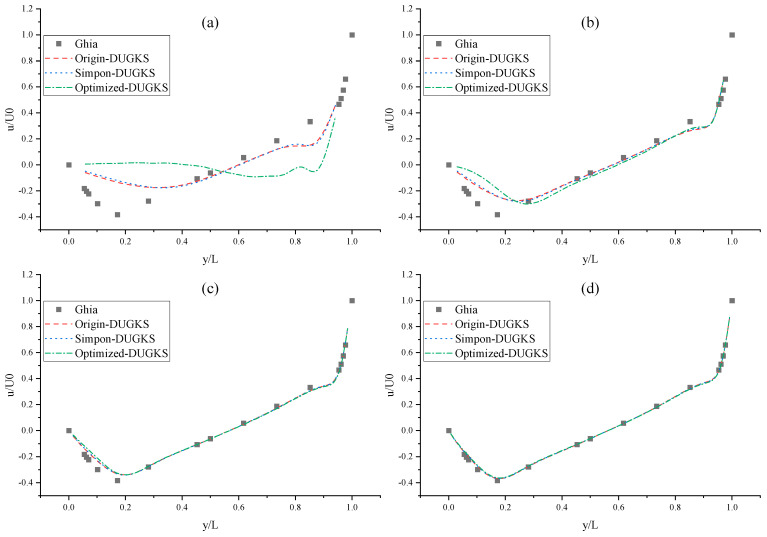
Horizontal velocity distribution of vertical central axis at Re = 1000 (CFL = 0.95). (**a**) N = 16, (**b**) N = 32, (**c**) N = 64, (**d**) N = 128.

**Figure 10:**
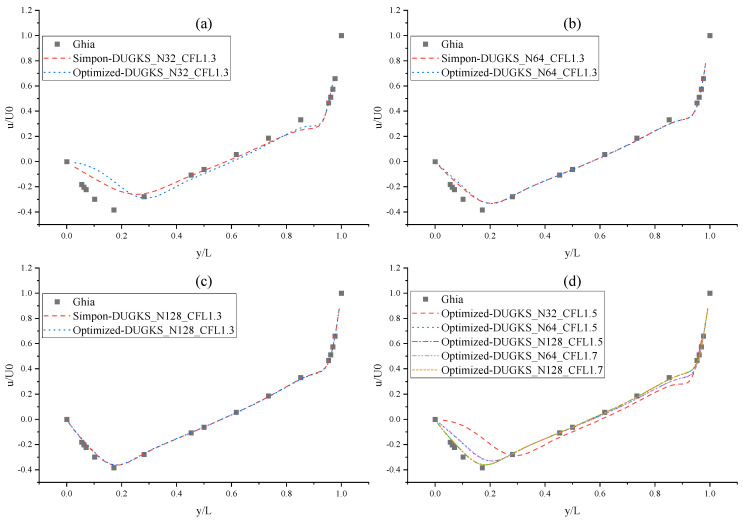
Horizontal velocity distribution of vertical central axis at Re = 1000. (**a**) N = 32, CFL = 1.3, (**b**) N = 64, CFL = 1.3, (**c**) N = 128, CFL = 1.3, (**d**) CFL = 1.5 & 1.7.

**Figure 11:**
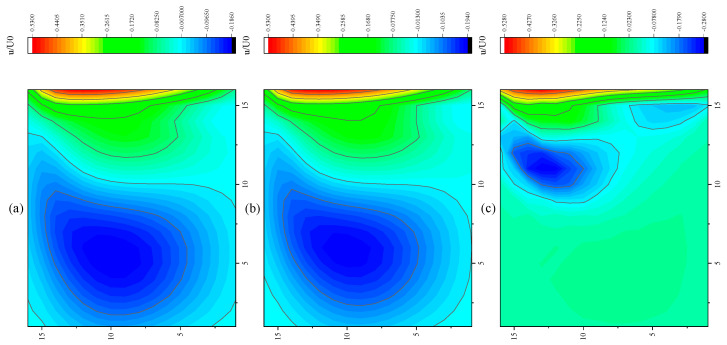
Horizontal velocity contours obtained by three methods at Re = 1000 with CFL = 0.95 and N*x* = N*y* = 16. (**a**) Original DUGKS, (**b**) Simpson–DUGKS, (**c**) optimized DUGKS.

**Figure 12:**
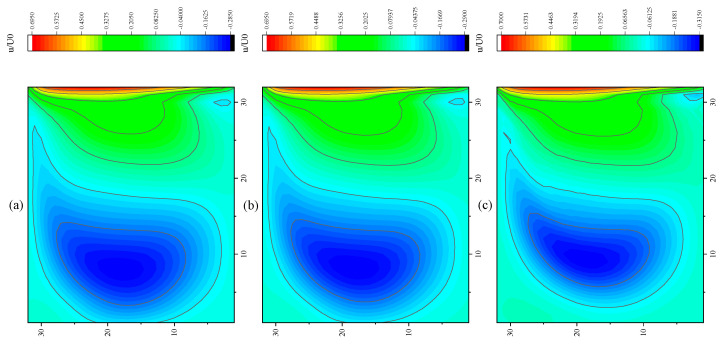
Horizontal velocity contours obtained by three methods at Re = 1000 with CFL = 0.95 and N*x* = N*y* = 32. (**a**) Original DUGKS, (**b**) Simpson–DUGKS, (**c**) optimized DUGKS.

**Figure 13:**
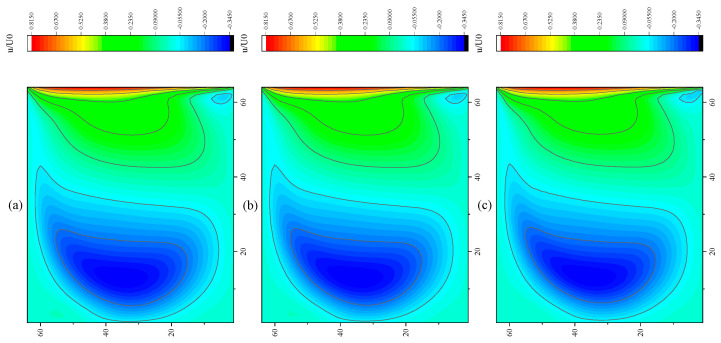
Horizontal velocity contours obtained by three methods at Re = 1000 with CFL = 0.95 and N*x* = N*y* = 64. (**a**) Original DUGKS, (**b**) Simpson–DUGKS, (**c**) optimized DUGKS.

**Figure 14:**
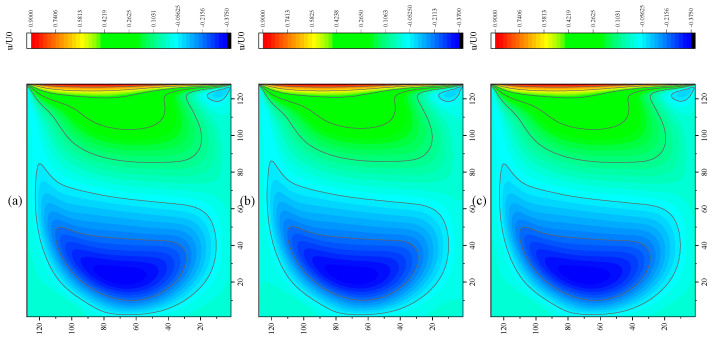
Horizontal velocity contours obtained by three methods at Re = 1000 with CFL = 0.95 and N*x* = N*y* = 128. (**a**) Original DUGKS, (**b**) Simpson–DUGKS, (**c**) optimized DUGKS.

**Figure 15:**
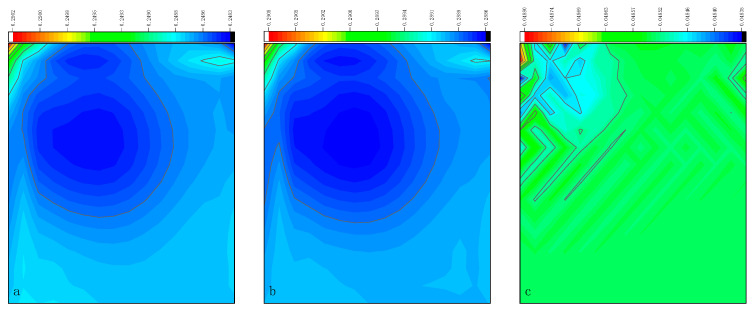
Pressure contours obtained by three methods at Re = 1000 with CFL = 0.95 and N*x* = N*y* = 16. (**a**) Original DUGKS, (**b**) Simpson–DUGKS, (**c**) optimized DUGKS.

**Figure 16:**
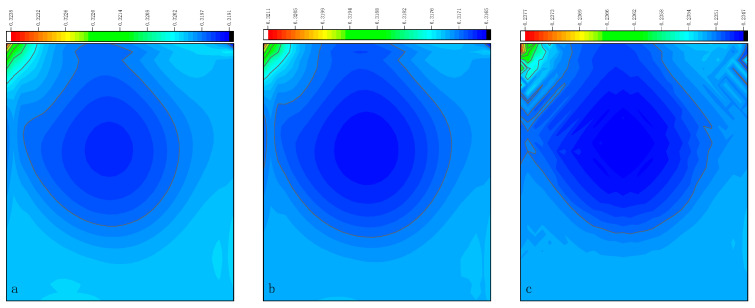
Pressure contours obtained by three methods at Re = 1000 with CFL = 0.95 and N*x* = N*y* = 32. (**a**) Original DUGKS, (**b**) Simpson–DUGKS, (**c**) optimized DUGKS.

**Figure 17:**
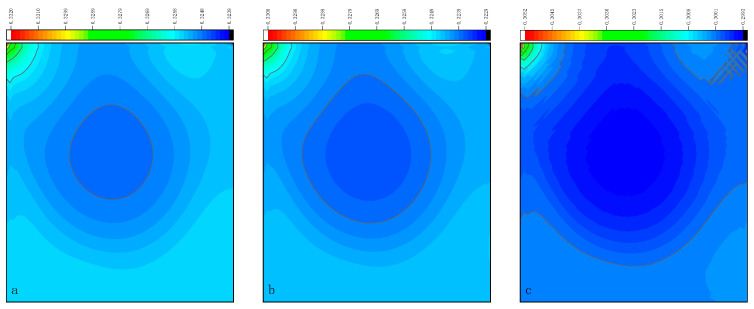
Pressure contours obtained by three methods at Re = 1000 with CFL = 0.95 and N*x* = N*y* = 64. (**a**) Original DUGKS, (**b**) Simpson–DUGKS, (**c**) optimized DUGKS.

**Figure 18:**
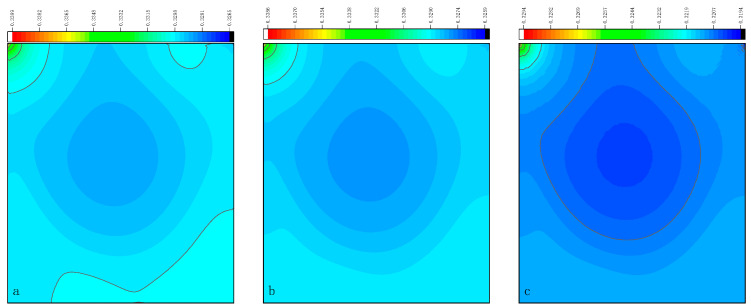
Pressure contours obtained by three methods at Re = 1000 with CFL = 0.95 and N*x* = N*y* = 128. (**a**) Original DUGKS, (**b**) Simpson–DUGKS, (**c**) optimized DUGKS.

The CPU time calculated by the three methods is shown in Table 9. Note that the symbol “-” indicates that the calculation does not converge, Δ1 represents the percentage increase in the calculation time of Simpson–DUGKS compared with the original DUGKS, and Δ2 represents the percentage decrease in the calculation time of optimized DUGKS compared with the original DUGKS. In terms of computational efficiency, compared with the original DUGKS, the computational time of the optimized DUGKS can be reduced by about 77% at most. It should be noted that, when CFL = 0.1 and N = 16, the calculation time of optimized DUGKS increases by about 40% compared with the original DUGKS. When CFL = 0.95 and N = 16, the calculation time of Simpson–DUGKS is reduced by about 59% compared with the original DUGKS. In terms of stability, the optimized DUGKS can still be computationally stable at large CFL numbers (CFL = 1.5 and 1.7). In terms of accuracy, the horizontal velocity of the vertical central axis obtained by the three methods is compared with the reference data, as shown in Figure 8, Figure 9 and Figure 10. It can be seen that the result of optimized DUGKS deviates more from the result of Ghia et al. [59], especially in the case of a small grid number. With CFL = 0.95, the horizontal velocity of the vertical central axis is shown in Figure 11, Figure 12, Figure 13 and Figure 14. With N = 64 and 128, the distribution of horizontal velocity obtained by the three methods is roughly similar, but with N = 16 and 32, the distribution of horizontal velocity obtained by optimized DUGKS is different from the other two methods, especially when N = 16, the error of horizontal velocity obtained by optimized DUGKS is obviously large. As shown in Figure 15, Figure 16, Figure 17 and Figure 18, the results of the optimized DUGKS contain some oscillations at the corner, while the solution of the original DUGKS and Simpson–DUGKS is smoother. What is more, the average density obtained by the Simpson–DUGKS is closest to the initial density (*ρ*_0_ = 1). The density error exists in the result obtained by the optimized DUGKS. The total number of points in the optimized DUGKS is the least. For the lid-driven cavity flow, it seems that each point plays an important role in the performance of numerical method. So, the minimum number of points may result in the large error.

**Figure 19:**
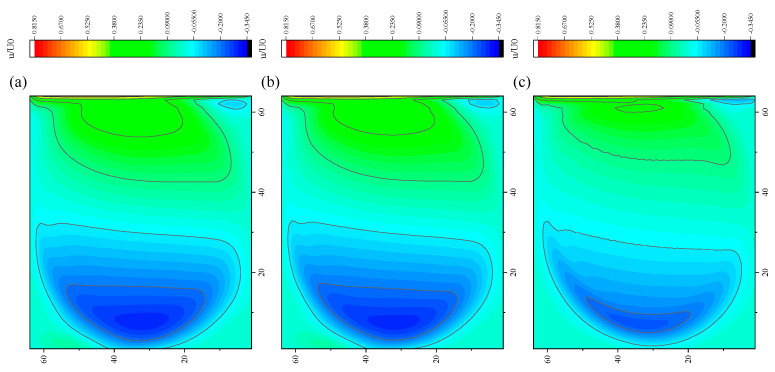
Horizontal velocity contours obtained by three methods at Re = 10,000 with CFL = 0.95 and N = 64. (**a**) Original DUGKS, (**b**) Simpson–DUGKS, (**c**) optimized DUGKS.

**Figure 20:**
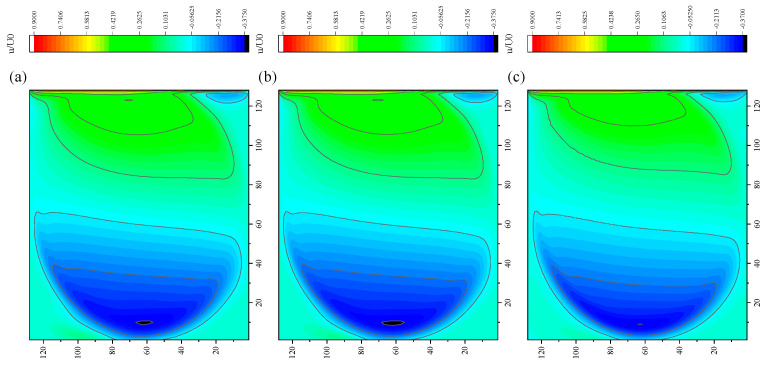
Horizontal velocity contours obtained by three methods at Re = 10,000 with CFL = 0.95 and N = 128. (**a**) Original DUGKS, (**b**) Simpson–DUGKS, (**c**) optimized DUGKS.

**Figure 21:**
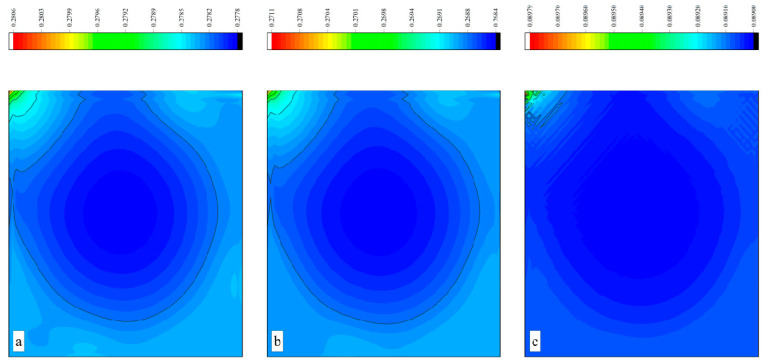
Pressure contours obtained by three methods at Re = 10,000 with CFL = 0.95 and N = 64. (**a**) Original DUGKS, (**b**) Simpson–DUGKS, (**c**) optimized DUGKS.

**Figure 22:**
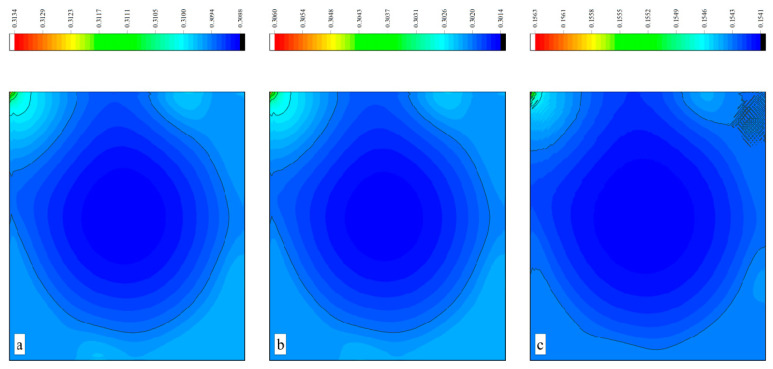
Pressure contours obtained by three methods at Re = 10,000 with CFL = 0.95 and N*x* = N*y* = 128. (**a**) Original DUGKS, (**b**) Simpson–DUGKS, (**c**) optimized DUGKS.

Furthermore, the performance of the three methods is tested under CFL = 0.1, 0.95, 1, 1.5, and 1.7 at Re = 10,000. The three methods cannot work when CFL = 1, 1.5, and 1.7. The three methods can converge when CFL = 0.1 and 0.95, and the calculation time to reach the convergence criterion is shown in Table 10. In terms of computational efficiency, compared with the original DUGKS, the computational time of the optimized DUGKS can be reduced by about 55% at most. In terms of stability, when CFL = 0.1 and N = 32, the optimized DUGKS cannot converge. As shown in Figure 19 and Figure 20, no non-physical oscillation phenomenon is found at the high Re number, and the velocity contours obtained by original DUGKS and Simpson–DUGKS are almost consistent, but the velocity contours obtained by optimized DUGKS are different, which seems to deviate from the reference results. As shown in Figure 21 and Figure 22, the results of the optimized DUGKS contain some oscillations at the corner, while the solution of the original DUGKS and Simpson–DUGKS is smoother.

The lattice BGK (LBGK) model and the multiple relaxation time (MRT) model, as popular standard LBM, have been successfully applied and well-accepted for incompressible NS solutions [60]. The pure stability of the LBGK, MRT, and Simpson–DUGKS method is evaluated without considering the accuracy of the results. To reach a steady-state solution at Re = 1000, the minimum required mesh resolution of the LBGK, MRT, and Simpson–DUGKS method is the 70 × 70, 20 × 20, 10 × 10 uniform mesh, respectively. On a uniform 128 × 128 mesh, the computations from the LBGK and MRT blow up at Re = 1900 and Re = 8000, respectively. However, the Simpson–DUGKS method works even at Re = 100,000. Clearly, in comparison with standard LBM, the Simpson–DUGKS method has super performance in stability.

Moreover, the average density obtained by the Simpson–DUGKS is closest to the initial density (*ρ*_0_ = 1). The density error exists in the result obtained by the optimized DUGKS. The reasons for the large density error can be explained as follows:

(1) Spatial and temporal truncation errors: optimized DUGKS can introduce truncation errors, which may accumulate and lead to significant density errors in specific flow regimes, especially when the flow has complex structures or high gradients.
(2) Dispersion and dissipation errors: Optimized DUGKS can suffer from dispersion and dissipation errors. In some flows, these errors might be amplified, causing the density waves to propagate at incorrect speeds or with distorted shapes. This can lead to a discrepancy between the numerical solution and the exact solution, manifesting as a large density error.
(3) Complex flow structures: flow with complex structures, such as those involving multiple vortices, can pose challenges for optimized DUGKS. The method might have difficulties in accurately resolving these intricate structures, leading to errors in the density field. For example, in a flow with contact discontinuities, the optimized DUGKS may not capture the sharp density gradients at the discontinuities, resulting in a smeared density profile and larger errors.

## 4. Conclusions

In this paper, the Simpson method is proposed to calculate the interface flux according to the distribution function at the node and the midpoint of the cell interface, and the optimized DUGKS and Simpson–DUGKS, considering the force term, are derived. Then, the original DUGKS, optimized DUGKS, and Simpson–DUGKS are simply compared and analyzed in theory. Finally, through numerical tests, the following conclusions are obtained:

In the numerical test of steady unidirectional flow (Couette flow and Poiseuille flow), the *L*_2_ errors calculated by the three methods are consistent, and the stability of the three methods is consistent under different CFL numbers. In terms of computational efficiency, among the three methods, optimized DUGKS has the least computational time, and Simpson–DUGKS has the most computational time, which also verifies the theoretical analysis and comparison. In more complex flows, some findings should be noted:

(1) In the numerical test of Taylor–Green vortex flow, the stability of the three methods is consistent under different grid numbers. Compared with the analytical solution of velocity, the *L*_2_ error of optimized DUGKS is the smallest under the same number of grids, but the *L*_2_ error of optimized DUGKS is the largest under the same number of grids compared with the analytical solution of density. In terms of computational efficiency, among the three methods, optimized DUGKS has the least computational time, while Simpson–DUGKS has the most computational time.
(2) In the numerical test of the lid-driven cavity flow, the calculation time of the optimized DUGKS can be reduced by about 77% at most, compared with the original DUGKS. It should be noted that, when CFL = 0.1 and N = 16, the calculation time of optimized DUGKS increases by about 40% compared with the original DUGKS. When CFL = 0.95 and N = 16, the calculation time of Simpson–DUGKS is reduced by about 59% compared with the original DUGKS. In terms of stability, the optimized DUGKS can still be computationally stable at large CFL numbers (CFL = 1.5 and 1.7). In terms of accuracy, the results of optimized DUGKS deviate more from the reference results, especially in the case of a small grid number. For example, when CFL = 0.95 and N = 16, the error of horizontal velocity contours obtained by optimized DUGKS is obviously large. This may be explained by the fact that the optimized DUGKS requires the fewest number of auxiliary points.

The guidelines on how to select appropriate DUGKS flux calculation methods are shown as follows: The optimized DUGKS is recommended for steady flow (Couette/Poiseuille flow, etc.) where efficiency is critical, which achieves 40–77% faster computation at large CFL. The Simpson–DUGKS is preferred for unsteady/vortical flow (Taylor–Green/cavity flow, etc.) requiring a high resolution of gradients, which reduces *L*_2_ errors by 30–50% with low grid resolution at moderate CFL, ensuring excellent stability.

## Figures and Tables

**Table 1:** CPU time, *L*_2_ error, and number of steps to reach converge at Re = 100.

CFL	N*x* = N*y*	*L*_2_ Error	Number of Steps	CPU Time (s)				
				Original DUGKS	Simpson– DUGKS	Optimized DUGKS	Δ1	Δ2
0.1	16	2.38 × 10^−5^	270,000	63.91	111.81	49.18	74.95%	23.05%
	32	4.55 × 10^−5^	477,000	462.34	817.19	322.38	76.75%	30.27%
	64	9.02 × 10^−5^	859,000	3818.79	6515.43	2408.6	70.62%	36.93%
	128	1.78 × 10^−4^	1,563,000	26,650.16	35,060.65	21,806.14	31.56%	18.18%
0.5	16	3.81 × 10^−6^	63,000	17.12	29.45	12.02	72.02%	29.79%
	32	8.16 × 10^−6^	112,000	111.58	211.32	78.73	89.39%	29.44%
	64	1.73 × 10^−5^	203,000	919.18	1871.22	541.88	103.57%	41.05%
	128	3.52 × 10^−5^	373,000	8172.24	10,395.84	4409.96	27.21%	46.04%
0.95	16	1.90 × 10^−6^	35,000	11.32	18.09	6.43	59.81%	43.20%
	32	3.68 × 10^−6^	63,000	72.59	122.96	44.57	69.39%	38.60%
	64	8.41 × 10^−6^	114,000	529.62	1090.94	303.46	105.99%	42.70%
	128	1.75 × 10^−5^	210,000	4107.69	6703.03	2447.27	63.18%	40.42%
1	16	1.42 × 10^−6^	34,000	11.16	16.49	6.66	47.76%	40.32%
	32	3.56 × 10^−6^	60,000	75.02	113.61	43.75	51.44%	41.68%
	64	7.81 × 10^−6^	109,000	499.07	1098.91	334.94	120.19%	32.89%
	128	1.71 × 10^−5^	200,000	3943.93	6306.43	2365.88	59.90%	40.01%
1.5	16	1.18 × 10^−6^	23,000	6.96	12.36	5.18	77.59%	25.57%
	32	2.61 × 10^−6^	41,000	48.11	82.77	33.65	72.04%	30.06%
	64	5.38 × 10^−6^	75,000	383.26	802.2	239.67	109.31%	37.47%
	128	1.08 × 10^−5^	139,000	2849.27	4691.96	1679.31	64.67%	41.06%
1.7	16	-	-	-	-	-	-	-
	32	7.44 × 10^−7^	21,000	6.32	12.37	3.85	95.73%	39.08%
	64	1.96 × 10^−6^	37,000	46.24	79.87	25.35	72.73%	45.18%
	128	4.64 × 10^−6^	67,000	342.42	698.74	180.01	104.06%	47.43%

**Table 2:** CPU time, *L*_2_ error, and number of steps to converge at Re = 10,000 with N*x* = N*y* = 128.

CFL	*L*_2_ Error	Number of Steps	CPU Time (s)				
			Original DUGKS	Simpson– DUGKS	Optimized DUGKS	Δ1	Δ2
0.95	1.878066 × 10^−3^	11,831,000	270,194.67	278,382.72	165,471.65	3.03%	38.76%
1.5	1.189824 × 10^−3^	8,060,000	199,466.71	202,131.9	172,020.05	1.32%	13.76%
1.7	1.049839 × 10^−3^	7,249,000	188,147.77	233,224.22	158,852.38	23.96%	15.57%

**Table 3:** CPU time, *L*_2_ error, and number of steps to reach convergence at Re = 100.

CFL	N*x* = N*y*	CPU Time (s)					*L*_2_ Error	Number of Steps
		Original DUGKS	Simpson– DUGKS	Optimized DUGKS	Δ1	Δ2		
0.1	16	100.79	149.77	75.44	48.60%	25.15%	0.102804	274,000
	32	720.16	1169.49	499.15	62.39%	30.69%	0.058279	485,000
	64	5426.69	7813.4	3637.99	43.98%	32.96%	0.030417	877,000
	128	37,932.5	51,146.37	28,019.24	34.84%	26.13%	0.015342	1,598,000
0.5	16	23.7	33.49	17.63	41.31%	25.61%	0.095353	64,000
	32	161.33	223.71	116.22	38.67%	27.96%	0.056502	113,000
	64	1324.72	1976.8	840.31	49.22%	36.57%	0.030133	207,000
	128	9188.03	13,185.13	6398.8	43.50%	30.36%	0.015423	380,000
0.95	16	12.93	20.25	9.91	56.61%	23.36%	0.096755	36,000
	32	87.2	124.37	65.94	42.63%	24.38%	0.056514	63,000
	64	661.76	1018.5	469.13	53.91%	29.11%	0.030074	116,000
	128	5390.39	7759.16	3656.11	43.94%	32.17%	0.015404	213,000
1	16	13.37	19.98	10.69	49.44%	20.04%	0.097037	34,000
	32	93.56	142.11	72.68	51.89%	22.32%	0.056554	61,000
	64	734.11	1155.93	489.51	57.46%	33.32%	0.030076	110,000
	128	5205.93	7473.29	3441.71	43.55%	33.89%	0.015403	204,000
1.5	16	10.03	15.51	7.04	54.64%	29.81%	0.100341	24,000
	32	68.14	109.34	44.25	60.46%	35.06%	0.057129	42,000
	64	522.65	829.57	319.33	58.72%	38.90%	0.030144	76,000
	128	3630.2	5254.6	2467.22	44.75%	32.04%	0.015396	141,000
1.7	16	9.64	14.83	5.97	53.84%	38.07%	0.101791	21,000
	32	66.03	103.21	41.76	56.31%	36.76%	0.057416	38,000
	64	464.12	730.12	303.73	57.31%	34.56%	0.030189	68,000
	128	-	-	-	-	-	-	-

**Table 4:** CPU time, *L*_2_ error, and number of steps to reach convergence at Re = 10,000 with CFL = 1.7 and N*x* = N*y* = 128.

CPU Time (s)					*L*_2_ Error	Number of Steps
Original DUGKS	Simpson–DUGKS	Optimized DUGKS	Δ1	Δ2		
249,234.44	255,189.79	138,468.52	2.39%	44.44%	0.01405448	7,455,000

**Table 5:** Convergence error and *L*_2_ error calculated by original DUGKS to 10,000 steps.

N*x* = N*y*	Convergence Error	*L*_2_ Error of Horizontal Velocity	*L*_2_ Error of Vertical Velocity	*L*_2_ Error of Density
16	3.796248 × 10^−3^	1.407493 × 10^−1^	1.390931 × 10^−1^	1.753841 × 10^−5^
32	9.123970 × 10^−4^	3.110016 × 10^−3^	3.103787 × 10^−3^	1.435340 × 10^−6^
64	2.206357 × 10^−4^	6.720014 × 10^−5^	6.717489 × 10^−5^	1.309586 × 10^−7^
128	5.624876 × 10^−5^	2.920428 × 10^−6^	2.920423 × 10^−6^	1.276418 × 10^−8^

**Table 6:** Convergence error and *L*_2_ error calculated by Simpson–DUGKS to 10,000 steps.

N*x* = N*y*	Convergence Error	*L*_2_ Error of Horizontal Velocity	*L*_2_ Error of Vertical Velocity	*L*_2_ Error of Density
16	4.106844 × 10^−3^	1.309639 × 10^−1^	1.308380 × 10^−1^	1.860117 × 10^−5^
32	9.011641 × 10^−4^	2.687649 × 10^−3^	2.684068 × 10^−3^	1.275103 × 10^−6^
64	2.099531 × 10^−4^	6.469734 × 10^−5^	6.463202 × 10^−5^	1.285961 × 10^−7^
128	5.535000 × 10^−5^	2.830797 × 10^−6^	2.830400 × 10^−6^	1.319192 × 10^−8^

**Table 7:** Convergence error and *L*_2_ error of optimized DUGKS calculation to 10,000 steps.

N*x* = N*y*	Convergence Error	*L*_2_ Error of Horizontal Velocity	*L*_2_ Error of Vertical Velocity	*L*_2_ Error of Density
16	1.216599 × 10^−3^	6.491338 × 10^−2^	5.887564 × 10^−2^	5.567707 × 10^−5^
32	3.288855 × 10^−4^	1.800740 × 10^−3^	1.774188 × 10^−3^	3.178361 × 10^−6^
64	1.920442 × 10^−4^	6.009158 × 10^−5^	5.993777 × 10^−5^	1.746339 × 10^−7^
128	5.446530 × 10^−5^	2.732364 × 10^−6^	2.731590 × 10^−6^	1.393446 × 10^−8^

**Table 8:** CPU time (s) calculated by three methods.

N*x* = N*y*	Original DUGKS	Simpson–DUGKS	Optimized DUGKS	Δ1	Δ2
16	4.89	5.96	4.32	22%	12%
32	13.68	17.58	11.03	29%	19%
64	47.45	61.52	37.01	30%	22%
128	183.57	236.9	138.77	29%	24%

**Table 9:** CPU time (s) to reach convergence calculated by three methods at Re = 1000.

CFL	N*x* = N*y*	Original DUGKS	Simpson–DUGKS	Optimized DUGKS	Δ1	Δ2
0.1	16	46.48	102.26	65.1	120%	−40%
	32	512.08	907.5	334.52	77%	35%
	64	3836.81	8557.54	2664.58	123%	31%
	128	31,612.86	82,837.44	23,141.43	162%	27%
0.95	16	67.17	27.73	16.61	−59%	75%
	32	62.41	84.73	55.25	36%	11%
	64	454.8	591.63	308.51	30%	32%
	128	3184.97	4371.63	2128.85	37%	33%
1.3	16	-	-	-	/	/
	32	-	80.09	67.03	/	/
	64	-	574.24	417.9	/	/
	128	-	4629.64	2677.79	/	/
1.5	16	-	-	-	/	/
	32	-	-	41.02	/	/
	64	-	-	218.79	/	/
	128	-	-	1241.96	/	/
1.7	16	-	-	-	/	/
	32	-	-	-	/	/
	64	-	-	196.97	/	/
	128	-	-	1090.08	/	/

**Table 10:** CPU time (s) to reach convergence at Re = 10,000.

CFL	N	Original DUGKS	Simpson–DUGKS	Optimized DUGKS	Δ1	Δ2
0.1	32	3503.67	7056.31	cannot converge	101%	/
	64	32,251.44	49,593.47	19513.29	54%	39%
	128	239,206.58	440,938.26	206,700.12	84%	14%
0.95	64	5997.67	7347.21	2963.74	23%	51%
	128	74,691.44	52,091.25	33,552.44	−30%	55%

## Data Availability

The data that supports the findings of this study are available within the article.

## Appendix A

In this paper, the calculation of interface flux is based on the distribution function $f(\xi_{\alpha },x,t^{n+1/2})$ at the time $t^{n+1/2}=t^{n}+h\;(h=\Delta t/2)$, which is abbreviated as $f^{n+1/2}$. The second-order Taylor expansion relative to time is as follows:

(A1) $$f^{n+1/2}=f^{n}+h\frac{\partial f^{n}}{\partial t}+\frac{h^{2}}{2}\frac{\partial^{2}f^{n}}{\partial t^{2}}+O(h^{3})$$

According to Equation (1), the partial derivative of the function *f* with respect to time is as follows:

(A2) $$\frac{\partial f}{\partial t}=-\xi_{\alpha }\cdot \nabla f+\Omega +S$$

Combining Equations (A1) and (A2),

(A3) $$\begin{array}{l} f^{n+1/2}=\left(f^{n}+\frac{h}{2}\Omega^{n}+\frac{h}{2}S^{n}\right)-h\xi_{\alpha }\cdot \nabla \left(f^{n}+\frac{h}{2}\Omega^{n}+\frac{h}{2}S^{n}\right)\\ +\frac{h^{2}}{2}\xi_{\alpha }\cdot \nabla \left[\xi_{\alpha }\cdot \nabla \left(f^{n}+\frac{h}{2}\Omega^{n}+\frac{h}{2}S^{n}\right)\right]+\frac{h}{2}\Omega^{n+1/2}+\frac{h}{2}S^{n+1/2}+O(h^{3}) \end{array}$$

Ignoring the second-order term in Equation (A3),

(A4) $$\underset{{\bar{f}}^{n+1/2}}{\underset{︸}{f^{n+1/2}-\frac{h}{2}\Omega^{n+1/2}-\frac{h}{2}S^{n+1/2}}}=\underset{{\bar{f}}^{+,n}}{\underset{︸}{\left(f^{n}+\frac{h}{2}\Omega^{n}+\frac{h}{2}S^{n}\right)}}-h\xi_{\alpha }\cdot \nabla \underset{{\bar{f}}^{+,n}}{\underset{︸}{\left(f^{n}+\frac{h}{2}\Omega^{n}+\frac{h}{2}S^{n}\right)}}$$

The distribution function at the cell interface center ***x****_b_* is as follows:

(A5) $${\bar{f}}_{b}^{n+1/2}={\bar{f}}_{b}^{+,n}-h\xi_{\alpha }\cdot \nabla {\bar{f}}_{b}^{+,n}$$

Equation (A5) corresponds to Equations (17) and (18).

The distribution function at the node ***x****_N_* is as follows

(A6) $${\bar{f}}_{N}^{n+1/2}={\bar{f}}_{N}^{+,n}-h\xi_{\alpha }\cdot \nabla {\bar{f}}_{N}^{+,n}$$

Equation (A6) corresponds to Equation (30).
